# Effect of flick application on pain level and duration of crying during infant vaccination

**DOI:** 10.1186/s13052-016-0218-y

**Published:** 2016-01-21

**Authors:** Esra Karaca Ciftci, Funda Kardas Ozdemir, Diler Aydın

**Affiliations:** Department of Child Health Nursing, Faculty of Health Sciences, Zirve University, 27260 Gaziantep, Turkey; Department of Nursing, Kars School of Health, Kafkas University, 36000, Kars, Turkey; Department of Nursing, Faculty of Health Science, Bandirma Onyedi Eylul University, 10200, Bandırma, Balıkesir Turkey

**Keywords:** Flick, Newborn, Vaccination, Pain, Nursing

## Abstract

**Background:**

The aim of the research is to determine the effect of flick application to reduce pain on pain level and duration of crying during vaccination.

**Method:**

This research was carried out on one-month-old babies in a family health center between March and June 2015. The babies coming for the second dose of Hepatitis B vaccine were divided into experiment and control groups. The babies in experiment group were flicked just before they were vaccinated. On the other hand, the babies in control group were vaccinated in usual way, with no other application. The pain level of babies in both groups was determined using “Neonatal Infant Pain Scale”. In addition, babies’ duration of crying was recorded.

**Results:**

In the study, it was detected that there was not a significant difference between pain score averages of babies in experiment and control groups (*p* > 0.05) before the application, however a significant difference in pain score average was detected during the application (*p* < 0.01) and after the application (*p* < 0.001). Babies’ duration of crying was compared and it was determined that babies in experiment group cried for shorter period, but no relevance was found (*p* > 0.05).

**Conclusions:**

Flick application at vaccination area could be used to reduce pain during vaccination at babies.

## Background

Vaccination is one of the most common painful procedures in infants. (1). Painful events such as vaccination is repeated and uncontrolled. Pain can cause short- and long-term effects in infants. (2). Its short-term effects include a delay in wound healing, a change in immunity system function, endocrine and biochemical changes, increased cortisol and catecholamine release, increased glucagon, growth hormone, aldosterone, rennin, and antidiuretic hormones, and decreased insulin secrection. They also include physiological changes such as apnea, bradycardia, tachycardia, skin color changes, hypertension, sweating palms, increased reased respiration rate and muscular tonicity, increased intracranial pressure and axygen intake, and behavioral changes, crying, ect (3). The studies have shown that uncontrolled pain experienced during early life has negative and long-term side effects, such as distress (4), and such experiences negatively affects the development of the central nervous system (5). It is therefore important that the number of painful stimuli be kept to a minimum, and that these stimuli are rendered less painful during this period for each procedure (6). The purpose in pediatric pain management is to reduce physiological and behavioral causes, to reduce intensity and duration of pain to minimum, to lessen the risk, and to give maximum benefits (7).

Nowadays various methods are used to reduce procedural pain in the newborn. Pharmacological and non-Pharmacological methods are used to relieve pain in newborn (2, 8). Non-pharmacological methods are alternatives for pain control, which implement small non-invasive attempts to reduce the pain. Position change, kangaroo care and touch, massage, teat giving, sucrose giving, breastfeeding, playing music, reducing distractions, and personalized developmental care are among these methods (2, 8, 9). In addition, methods such as distracting (10), cold application (11), massage (12, 13), vibration (11, 14) injection technique, and manual pressure application (15, 16) are used to reduce pain during invasive attempts.

Flick application at the injection area could be used to control pain during childhood (17). It was proven that applying manual pressure to the injection area (having a similar effect to the flick method) reduced injection pain (15, 16, 18). However, a response to the flick method had not been detected yet.

The purpose of this research was to examine the effect of flick application on reducing pain and duration of crying in infants during vaccination.

## Methods

### Study design

This study was a randomized controlled clinical trial conducted during routine vaccine injections.

### Setting and samples

The research was conducted at a family health center (FHC) in the east of Turkey. According to records, this heath center had provided service for 11780 people and 352 infants during 2014. Data of the research was collected during working hours between March and June 2015. The research was carried out in vaccination room. The infants, whose mothers accepted to take part in the research, were brought to FHC at the end of first month for the second dose of Hepatitis B vaccine, and they were flicked at injection area just before they were vaccinated.

Population of the research includes all the infants who applied for second dose of Hepatitis B vaccine to FHC between March and June 2015.

Sample of the research includes the infants who applied for second dose of Hepatitis B vaccine to FHC during the research period and who met following criteria. Sample of the research includes 70 infants, 35 for experimental group, and 35 for control group. In the power analysis performed to determine the sample size, the calculated sample size was found to be 70 infants in order to achieve a 0.80 power in the test, at a significance level of 0.05 with a medium level of effect. Sample groups were randomly selected during the research period; the first infant arriving for vaccination was registered in the control group, whereas the second infant was registered in the experimental group, and so on. Each group included 35 infants.

#### Inclusion Criteria

The sample group, which was formed after written approval of parents, was selected by taking some criteria into consideration. Some of the criteria for infants are that gestational age is 37-42 weeks, term and healthy, birth weight is 2500 gr and above, 30-42 days old after birth, no previous treatment experience of intramuscular injection, no pain-killing medicine before vaccination.

### Data Collecting Tools

The question form and Neonatal Infant Pain Scale(NIPS) were used in data collection and the infants’ responses to the procedure were video recorded.

### Question Form

This form, prepared by the researchers, based on relevant literature, comprised questions to collect participants’ demographic data, such as, gender, age, weight, duration of crying (1, 6, 10, 11, 18, 19). The form was filled out during face-to-face interviews held with the parents of the infants, who had volunteered to participate in the study.

### Neonatal Infant pain Scale (NIPS)

NIPS was developed to evaluate neonate infants’ behavioral pain response before, during, and after injection attempt by Lawrance et al., (20). Validity and reliability for Turkish were completed by Akdovan in 1999 (21). Neonate infant pain scale is formed by 6 behavioral sections including facial expression, crying, breathing style, arm and leg movements, and being woken up. Crying has 3 points (0-1-2), and other behaviors have 2 points (0-1). Total score is between 0 and 7. Higher score shows that the pain is more intense. Akdovan found Cronbah alpha scores as 0,83-0,83-0,86 in his study (19, 21) Internal consistency Cronbach Alpha score of this scale was detected 0.81 before, 0.76 during, and 0.78 after operation.

### Procedure

Subsequently, research was carried out with those infants whose parents were willing to participate after informed consents were obtained from them. Data collection forms were completed by researchers. Neonates in both control and experimental group were vaccinated by the same staff working in the family health center (FHC). All infants were awake, had clean diapers at the time of injection, and their parents were in the procedure room.

The infants in the control group were regularly vaccinated, without any other attempt in FHC, and they were videotaped before, during, and after the vaccination. During the vaccination procedure, the parents of infants in both groups were allowed to calm their babies by touching and talking to them, but not to feed and do anything that would distract the infant’s attention, including giving them toys, showing them a dummy, or clapping.

### Routine application of Hepatitis B vaccine

The infant was laid back, and the vaccination area was marked. The Hepatitis B vaccine was intramuscularly (IM) injected. The injection site was located at the ventrolateral section of the vastus lateralis muscle, which is in the upper 1/3 part of the thigh. The vaccination area was disinfected with alcohol, and the vaccine was injected into the upper 1/3 section of the vastus lateralis muscle at 90-degree angle.

Each infant in the experimental group was flicked once at the vaccination area (vastus lateralis) prior Hepatitis B vaccination.

### Flick application

**Fig. 1:**
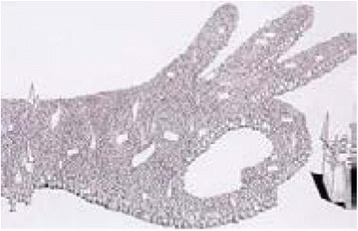
Flick application

The Nurse determined the vaccination area and disinfected it using cotton with 70 % alcohol. The muscle was held with the nurse’s left hand, and the vaccination area was flicked with the right hand. The flick was given as follows: the thumb was placed on the nail of middle finger, then the vaccination area was stimulated with a quick tap using upper nail part of middle finger (Fig. 1).

It was ensured that the hands of flicking staff were warm, and nails were clipped in order not to harm the infant. The staff waited to start the procedure until the infant calm down, if it was anxious; the procedure was not initiated if the infants were crying.

Infants in both the experimental and control group were videotaped before, during, and after vaccination until they stopped crying. After vaccination, filming was continued for 1 min, and, following ethical principles, the mothers were allowed to comfort their babies that continued crying for >1 min. Video records lasted about 1.5 - 2 min. The infant’s whole body and the crying sounds, which were indicative of pain, (22) were video recorded using a Canon Ixus 75 7.1 MP digital video camera.

The procedure was recorded using a video camera. Video records were evaluated independently by two specialist observers (pediatric specialist nurse, neonatal doctor). Observers were not informed which newborn belonged to the control group and which newborn belonged to the treatment group. After records were completed, they were evaluated twice by two experts at different times. Observers scored the NIPS by evaluating the pain experienced by the newborns. The concordance coefficient was calculated between observers. Average Neonatal Infant Pain Scale (NIPS) scores were calculated at the end of evaluations. Observers’ evaluations were found consistent (for each of 6 categories of NIPS, Kappa observers consistency test gives the result in range of 0.48 and 0.71).

The duration of neonates’ crying (sec) was recorded from the video records.

### Data Analysis

Data from the research were evaluated in SPSS (Statistical Package for Social Sciences) 13.0 software package. Percentage distribution, average, standard deviation, chi-square test, cronbach alpha coefficient of consistence, chi-square in comparing the groups, Independent Sample t test, and One-way Analysis of Variance were used in analysis of research results. *P* < 0.05 was considered significant in all analyses.

### Ethical Considerations

The study was approved by the local institution based on regulation No 2015.3/1 by the Ethical Board of Kafkas University, Medicine School. As responses should be given voluntarily in all researches, whereby information is obtained, it was ensured that the parents of the infants to be included in the study were volunteers. Moreover, the parents of the infants were informed of the aim and protocol of the research (why the babies were recorded, and for what purpose these recordings were going to be used), and both their written and oral consents (informed consent principle) were received (23). All infants’ parents agreed to fill out the question form and were aware that the video recording was going to be short and that their babies were not going to be imposed with any extra burden.

## Results

Table 1 includes distribution of infants in experimental and control groups according to their descriptive features. It was determined that 51.4 % of infants in experimental group, and 54.3 % of infants in control group are male. Statistically, no significant difference between experimental and control groups was found in terms of gender of infants (x^2^ = 3.131, df = 1, *p* > 0.05). In the study, it was detected that gestational age was 39.86 ± 0.61 weeks, average weight was 3337.8 ± 444.7 grams, and height was 53.25 ± 2.03 cm for infants in experimental group, and that gestational age was 39.74 ± 0.74 weeks, average weight was 3496.5 ± 508.2 grams, and height was 53.60 ± 1.24 cm for infants in control group. When experimental and control groups were compared in other descriptive features of infants, no significant difference was found between groups in terms of weight, height, and gestational age (respectively t = 0.889, *p* > 0.05, t = 1.372, *p* > 0.05, t = 0.683, *p* > 0.05).

**Table 1:** Comparison of Control and Experimental Groups According to the Newborn’s Descriptive Characteristics

Features	Control Group		Experimental Group		Total		Test and p
	n	%	n	%	n	%	
Gender							
Female	16	45.7	17	48.6	33	47.1	*X* ^*2*^ * = 3.131*
Male	19	54.3	18	51.4	37	52.8	*df = 1, p > 0.05*
Gestational age (week)^a^	39.74 ± 0.74		39.86 ± 0.61		39.80 ± 0.67		*t = 0.854, p > 0.05*
Weight (g)^a^	3496.5 ± 508.2		3337.8 ± 444.7		3417,2 ± 476.5		*t = 1.372, p > 0.05*
Height (cm)^a^	53.60 ± 1.24		53.25 ± 2.03		53.43 ± 1.63		*t = 0.683, p > 0.05*

^a^ Average (SD)

In Table 2, average NIPS scores of experimental and control groups before, during and after vaccination and comparison between groups are shown. Pain score averages of infants in experimental group were as follows: before 0.23 ± 1.05, during 3.01 ± 2.09, and after vaccination 1.04 ± 1.99. In control group, it was detected as follows: before 0.52 ± 1.46, during 5.43 ± 2.47, and after vaccination 3.39 ± 2.24.

**Table 2:** Inter-Group Comparison of NIPS Score Average in Experimental and Control Groups in terms of Operation Time

	NIPS score averages			
Groups	Before	During	After	Test and p^a^
	X ± SS	X ± SS	X ± SS	
Experimental	0.23 ± 1.05	3.01 ± 2.09	1.04 ± 1.99	*F:23.485, p < 0.001*
Control	0.52 ± 1.46	5.43 ± 2.47	4.39 ± 2.24	*F:39.227, p < 0.001*
Test and p^b^	*t:1.156, p > 0.05*	*t:3.748, p < 0.01*	*t: 5.890, p < 0.001*	

^a^ One-way analysis of variance in iterative measurement

^b^ T test in dependent groups

It was detected that, in experimental group, pain scores were lower than control group before, during, and after operation. Infants’ pain score average differences before, during, and after operation were found statistically significant. Similarly, pain score average differences of infants in experimental and control group before, during, and after operation were found statistically significant.

In addition, in experimental and control groups, pain score average comparison within group before, during, and after operation was carried out, and it was detected that there was no significant difference in pain score average before operation (*p* > 0.05), but there was significant difference in pain score average during (*p* < 0.01), and after (*p* < 0.001) operation.

It was determined that average duration of crying of infants in experimental group was 35.64 ± 16.26, that of control group was 42.99 ± 20.56 seconds. Duration of crying of infants in experimental group was found shorter than that of control group. Difference between duration of crying of infants in experimental group and that of control group was not found statistically significant (*p* > 0.05) (Table 3).

**Table 3:** Comparison of Duration of Crying of Infants in Experimental and Control Groups

	Control Group	Experimental Group	Test and p
Duration of Crying (sec.)	42.99 ± 20.56	35.64 ± 16.26	*t=1,681, p > 0.05*

## Discussion

Pain in neonates is a complicated event, which is frequent and difficult to evaluate. It was believed that neonates and infants do not feel any pain, whereas numerous studies have shown that they feel more pain than children and adults (24). Nowadays various methods are used to reduce procedural pain in the newborn. Some nonpharmacological methods of stopping pain are touching, giving position, skin stimulation, heat and cold application, teat giving, breastfeeding, kangaroo care, and sucrose giving (25).

This study was conducted with an objective of providing an alternative to other prevalent nonpharmacological methods and to define the effect of flick application on pain during vaccination. The results of this study indicated that there was no significant difference in mean pain score between infants in the experimental and control groups before vaccination (*p* > 0.05), but there was a significant difference in mean pain score during (*p* < 0.01) and after the procedure (*p* < 0.001).

It is identified that flick application is among the effective methods for controlling pain during childhood (17). In concurrence with other studies, we found that flick application was effective in reducing pain during vaccination. The effect of manual pressure application, which has a similar effect to flick application, has also been examined in some studies. In the studies of Yavuz (2011), Barnhill et al (1996), Chung and Wong (2002), it was detected that manual pressure to the injection area prior to vaccination reduces injection pain (15, 16, 18). Furthermore, in the study of Uğurlu (2011), in which the effect of massage during vaccination was studied, it was observed that the pain scores of infants in an experimental group were lower than those in a control group during and after the operation. In the light of these results, it is suggested that massage is effective in reducing pain, and as the massage continues, the level of pain decreases (12).

The reduction of pain through skin stimulation is based on the Gate Control Theory, which was suggested by Wall and Melzack in 1965, and is still an accepted theory (26). In this theory, the presence and severity of pain depends on the passage of neurological stimulations and pain signals are transported on small fibers. The large fibers are stimulated through massage, rubbing, and pressure and the small fibers are prevented from transporting signals. As there are numerous large fibers in the skin, most touching stimulations have the potential to reduce pain (13, 27, 28). This theory explains the possibility that manual pressure and massage can reduce pain. As flick application conveys a similar stimulus, its effect on reducing pain can also be explained by Gate Control Theory.

According to a literature survey, research concerning the effect of flick application on reducing pain during vaccination has not yet been conducted in Türkiye or worldwide. The current study is the first to investigate this topic. Crying is an important indicator of pain for infants as it is their only means of communication and, among the behavioral methods, it is accepted as a practical method to evaluate the severity and duration of pain (2, 29).

In the current study, no statistical significance was detected regarding duration of crying between the experimental and control groups; however it was noted that infants in the experimental group cried for shorter periods than those in the control group. In the study of Uğurlu and Başbakkal (2011), similarly, it was found that infants in an experimental group cried for shorter time-periods than those in a control group (12). In the same study, a significant difference in average duration of crying was detected between the control and experimental groups (*p* < 0.05). In the study of Razek and El-Dain (2009), in which the effect of breastfeeding on reducing vaccine pain was studied, total duration of crying was 125 ± 12 sec for infants in an experimental group and 148 ± 13 sec for infants in a control group (30).

## Conclusion

Results indicated that the severity of pain was decreased for infants who were flicked on the vaccination area before immunization; thus, flicking before vaccination is probably effective in reducing pain and decreasing the duration of infant crying during vaccination in primary care health institutions. Finally, flicking before vaccination can be an easy to perform, guick, safe and inexpensive “add on” to other techniques in the management of vaccination pain. Furthermore, it is suggested that this study should also be performed in older groups and with larger sample sizes.
